# Lipoproteins of slow-growing Mycobacteria carry three fatty acids and are *N*-acylated by Apolipoprotein *N*-Acyltransferase BCG_2070c

**DOI:** 10.1186/1471-2180-13-223

**Published:** 2013-10-05

**Authors:** Juliane K Brülle, Andreas Tschumi, Peter Sander

**Affiliations:** 1 Institute of Medical Microbiology, University of Zurich, Gloriastrasse 30/32, CH-8006, Zurich, Switzerland; 2 National Reference Laboratory for Mycobacteria (NRLM), Gloriastrasse 30, CH-8006, Zurich, Switzerland

**Keywords:** Mycobacteria, Tuberculosis, Lipoprotein, Lipidation, Apolipoprotein *N*-acyltransferase

## Abstract

**Background:**

Lipoproteins are virulence factors of *Mycobacterium tuberculosis*. Bacterial lipoproteins are modified by the consecutive action of preprolipoprotein diacylglyceryl transferase (Lgt), prolipoprotein signal peptidase (LspA) and apolipoprotein *N*- acyltransferase (Lnt) leading to the formation of mature triacylated lipoproteins. Lnt homologues are found in Gram-negative and high GC-rich Gram-positive, but not in low GC-rich Gram-positive bacteria, although *N*-acylation is observed. In fast-growing *Mycobacterium smegmatis*, the molecular structure of the lipid modification of lipoproteins was resolved recently as a diacylglyceryl residue carrying ester-bound palmitic acid and ester-bound tuberculostearic acid and an additional amide-bound palmitic acid.

**Results:**

We exploit the vaccine strain *Mycobacterium bovis* BCG as model organism to investigate lipoprotein modifications in slow-growing mycobacteria. Using *Escherichia coli* Lnt as a query in BLASTp search, we identified BCG_2070c and BCG_2279c as putative *lnt* genes in *M. bovis* BCG. Lipoproteins LprF, LpqH, LpqL and LppX were expressed in *M. bovis* BCG and BCG_2070c *lnt* knock-out mutant and lipid modifications were analyzed at molecular level by matrix-assisted laser desorption ionization time-of-flight/time-of-flight analysis. Lipoprotein *N*-acylation was observed in wildtype but not in BCG_2070c mutants. Lipoprotein *N*- acylation with palmitoyl and tuberculostearyl residues was observed.

**Conclusions:**

Lipoproteins are triacylated in slow-growing mycobacteria. BCG_2070c encodes a functional Lnt in *M. bovis* BCG. We identified mycobacteria-specific tuberculostearic acid as further substrate for *N*-acylation in slow-growing mycobacteria.

## Background

Proteins posttranslationally modified by covalent lipid attachment are present in eukaryal and bacterial organisms. In bacteria, 1–3% of the genome encode for lipoproteins. Bacterial lipoproteins are anchored in the membrane surface where they fulfill various cellular functions, ranging from cell wall integrity, secretion, nutrient uptake, environmental signaling to virulence [1-3].

Lipoproteins from Gram-positive and Gram-negative bacteria are synthesized in the cytosol as preprolipoproteins and are translocated across the cytoplasmic membrane via the Sec- or Twin arginine translocation (Tat) system [4]. Lipoprotein signal sequences terminate in a highly conserved lipobox motif consisting of four amino acids (LVI/ASTVI/GAS/C) [2]. Processing of lipoprotein precursors into mature forms takes place at the outer leaflet of the cytoplasmic membrane and is accomplished by the sequential action of three enzymes attacking the conserved cysteine in the lipobox: 1) the phosphatidylglycerol:pre-prolipoprotein diacylglyceryl transferase (Lgt) attaches a diacylglyceryl residue to the cysteine via thioether linkage [5], 2) the prolipoprotein signal peptidase (LspA) cleaves off the signal peptide and 3) apolipoprotein *N*-acyltransferase (Lnt) acylates the N-terminal cysteine residue at its free amino group [1,6,7]. In proteobacteria, *N*-acylation of lipoproteins is a prerequisite for the transport to the outer membrane by the Lol system [8,9].

Lgt and LspA are universally present in Gram-positive and Gram-negative bacteria [10]. The gene encoding Lnt was originally identified in the Gram-negative bacterium *Salmonella enterica* sv. Typhimurium and is conserved in proteobacteria. The Lnt structure and function are well studied in *Escherichia coli*[11]. Contrary to the long held assumption that *lnt* is restricted to Gram-negative bacteria [10]*lnt* homologues are also present in high GC-rich Gram-positive bacteria. In the fast-growing, saprophytic mycobacterial model organism *Mycobacterium smegmatis*, Lnt-dependent *N*-acylation was demonstrated and the lipid moiety of lipoproteins has been resolved at molecular level. *M. smegmatis* lipoproteins are modified with a thioether-linked diacylglyceryl residue composed of ester-linked palmitic acid and ester-linked tuberculostearic acid and an additional palmitic acid amide-linked to the α-amino group of the conserved cysteine. Diacylglycerol modification and signal peptide cleavage are prerequisites for *N*-acylation [12,13]. Secreted proteins, among them lipoproteins often are modified by glycosylation. *O*-glycosylation in mycobacteria occurs through a stepwise process depending on at least a protein mannosyl tranferase (PMT) performing the initial mannosylation step and a α1-2 mannosyl tranferase realizing the subsequent elongation of the mannosyl chains. Recently, PMT enzyme responsible for the initial attachment of mannose residue to the protein was identified [14].

In addition to *M. smegmatis*, *N*-acyltransferase activity by Lnt homologues was shown in two other high GC-rich Gram-positive bacteria, namely *Streptomyces scabies*[15] and *Corynebacterium glutamicum*[16]. Recent mass spectrometry analyses of lipoproteins in low GC-rich Gram-positive bacteria (firmicutes and mollicutes) provided evidence that *N*-acylation also occurs in these bacterial species, however, no obvious *lnt*-like gene has been identified to date [17-21]. Instead, biochemical analysis identified two new lipoprotein structures, the “*N*-acetyl” and the “peptidyl” lipoprotein structure. These novel structures strongly suggest the presence of yet to be identified key enzymes involved in bacterial lipoprotein biosynthesis [22].

Most pathogenic mycobacteria belong to the group of slow-growing mycobacteria, including *Mycobacterium leprae*, the causative agent of leprosy and the members of the *Mycobacterium tuberculosis* complex (e.g. *M. tuberculosis, Mycobacterium africanum, Mycobacterium cannetti, Mycobacterium bovis*). *Mycobacterium tuberculosis* is the causative agent of human tuberculosis, a major cause of death around the world (http://www.who.int/tb/publications/factsheets/en/index.html). Elimination of tuberculosis requires an improved understanding of the host, the pathogen and their interaction for the development of better, more effective drugs and vaccines. Lipoprotein biogenesis is a major virulence factor of *M. tuberculosis*[23,24]. Moreover, lipoproteins evidently meet pathogen-associated molecular patterns (PAMPs) criteria and are well detected by innate immune recognition mechanisms [25]. *M. tuberculosis* lipoproteins are major antigens and trigger the activation of cellular and humoral immune responses to mycobacteria. Lipoproteins are potent agonists of toll-like receptor 2 (TLR2) which upon long term stimulation has been associated with the down regulation or deviation of the immune response. TLR2 agonist activity has been demonstrated for several *M. tuberculosis* lipoproteins including LpqH, LprA, LprG and PstSI [26,27]. Recently, it was reported that mycobacteria generate and release membrane vesicles (MVs) [28]. Strikingly, MVs from pathogenic mycobacteria as compared to non-pathogenic mycobacteria are enriched in lipoproteins, some of them well known TLR2 agonists. MVs produced a severe TLR2 dependent inflammatory response in vitro and in vivo [28]. Investigations regarding the vaccine potential of MVs from pathogenic mycobacteria elicited a mixed cellular and humoral immune response. This suggests a vaccine potential of MVs and their lipoproteins against *M. tuberculosis*.

Even though research on lipoproteins in fast-growing mycobacteria contributed to the knowledge of lipoprotein biosynthesis and modification, there is scarcely known anything about lipoprotein modifications and their chemical structures in slow-growing mycobacteria. *Mycobacterium bovis* bacille Calmette Guerin (BCG) is derived from virulent *M. bovis*, the causative agent of bovine tuberculosis. The genome of *M. bovis* BCG is highly similar to the *M. tuberculosis* genome (>99.5% sequence identity) [29]. *M. bovis* BCG was first used in 1921 as a live vaccine against tuberculosis. Since then four billion doses have been applied to humans. Still today it is the only licensed tuberculosis vaccine, despite its incomplete protective efficacy, particular against adult lung tuberculosis [30].

Concerning the presence of open reading frames (ORFs) encoding lipoprotein modifying enzymes, both genomes of *M. tuberculosis* and *M. bovis* BCG Pasteur reveal a single ORF encoding Lgt (Rv1614, BCG_1652) and a single ORF encoding LspA (Rv1539, BCG_1591). Two ORFs encoding Lnt are found in *M. bovis* BCG (BCG_2070c, BCG_2279c). BCG_2070c (which is identical to *M. tuberculosis* Rv2051c = *ppm1*) is a two domain protein with a conserved apolipoprotein-*N*-acyltransferase and a Ppm-like domain. BCG_2279c shows conserved apolipoprotein-*N*-acyltransferase domain and exhibits considerable homology to *E. coli* Lnt. In *M. tuberculosis*, the corresponding open reading frame is split into two, Rv2262c and Rv2261c. In our previous analysis [12], these may have escaped our attention, since split. Only upon completion of the *M. bov*is BCG sequence the homology to Lnt became apparent. Due to this polymorphism in the second *M. tuberculosis* putative Lnt ORF, we focussed our studies on lipoproteins and lipoprotein synthesis in slow-growing mycobacteria on the vaccine strain *M. bovis* BCG. Prediction of lipoproteins in *M. tuberculosis* complex using DOLOP database suggests the presence of 50 potential lipoproteins of the approximately 4000 ORFs [2]. However, the existence of twice as many lipoproteins has been discussed [1].

In this study, we show that lipoproteins are triacylated in slow-growing *M. bovis* BCG. We demonstrate apolipoprotein *N*-acyltransferase acitivity and by targeted gene deletion identify BCG_2070c as a functional Lnt. We give structural information about the lipid modification of four mycobacterial lipoproteins, LprF, LpqH, LpqL and LppX. Hereby mycobacteria-specific tuberculostearic acid is identified as a further substrate for *N*-acylation.

## Methods

### Bacterial strains and growth conditions

*Mycobacterium bovis* BCG Pasteur strains were cultivated in Middlebrook 7H9 medium or on Middlebrook 7H10 agar enriched with oleic acid albumin dextrose (OADC, Difco). Liquid broth was supplemented with 0.05% of Tween 80 to avoid clumping. If necessary, the appropriate antibiotic was added at the following concentration: 5 μg ml^-1^ gentamicin, 100 μg ml^-1^ streptomycin, 25 μg ml^-1^ hygromycin. Strains used in this study were *M. bovis* BCG SmR (further referred to as *M. bovis* BCG or parental strain) [31], a streptomycin resistant derivative of *M. bovis* BCG Pasteur 1173P2, Δ*lnt* = *M. bovis* BCG SmR *lnt* knock out mutant in BCG_2070c and Δ*lnt*-*lnt*BCG_2070c = *M. bovis* BCG SmR *lnt* knock out mutant in BCG_2070c transformed with complementing vector pMV361-hyg-*lnt*BCG_2070c.

### Disruption of *lnt* in *M. bovis BCG*

A 1.9 kbp *Mlu*I/*Nsi*I fragment of *M. bovis* BCG from position 2296156 to 2294306 comprising the 5’*lnt* flanking sequence and a 2.8 kbp *SnaB*I/*Mlu*I fragment from position 2292652 to 2289856 comprising the 3’*lnt* flanking sequence of the *lnt* domain of BCG_2070c were PCR amplified using genomic DNA from *M. bovis* BCG Pasteur and cloned into vector pMCS5-rpsL-hyg with the respective enzymes resulting in knock-out vector pMCS5-rpsL-hyg-Δ*lnt*BCG. This way, we deleted a 1.6 kbp of the Lnt domain without introducing a frameshift or any other deletion to the Ppm synthase domain. The *lnt*BCG allele was deleted in the *M. bovis* BCG SmR chromosome as described previously [31,32] and confirmed by Southern blot analysis with 0.2 kbp *Sal*I *lnt* downstream probe. For complementation with *M. bovis* BCG BCG_2070c a 6.3 kbp fragment from *M. bovis* BCG from position 2289839 to 2296178 spanning the entire *lnt* gene was cloned into pGEM-T Easy (Promega) to result in pGEM-T Easy-*lnt*BCG_2070c and subsequently subcloned as a 6.3 kbp *Eco*RI fragment into the *Hpa*I site of plasmid pMV361-hyg [33] to result in pMV361-hyg- *lnt*BCG_2070c. Complementation was confirmed by Southern blot analyses with 0.2 kbp *Kpn*I/*Hind*III *lnt*BCG_2070c upstream probe.

### Expression of Lipoproteins LprF, LpqH, LpqL and LppX

Plasmid pMV261-Gm, a derivative of pMV261 shuttle vector, is able to replicate in *E. coli* as well as in mycobacteria [34]. *LprF*[13], *lpqH*, *lpqL* and *lppX*[12] were amplified by PCR from *M. tuberculosis* genomic DNA and fused to the *M. tuberculosis* 19 kDa promoter. The target proteins and 19 kDa promoter are identical between *M. tuberculosis* and *M. bovis* BCG. Sequences encoding a hemagglutinin and a hexa- histidine epitope were fused to the 3’ part of each gene to facilitate subsequent purification and detection on Western blot. The insert was cloned into the *EcoR*I site of pMV261-Gm to result in pMV261-Gm-LprF, pMV261-Gm-LpqH, pMV261-Gm-LpqL and pMV261-Gm-LppX. Subsequently plasmids were transformed into BCG parental strain, Δ*lnt* and Δ*lnt*-*lnt*BCG_2070c.

### Preparation of cell extracts and Western blot analysis

Bacteria from 1-liter cultures were harvested and resuspended in phosphate-buffered saline containing Complete EDTA-free tablets (Roche) to inhibit protein degradation. Cells were lysed by three French Press cycles (American Instrument Co.) at 1.1 x 10^6^ Pa. Extracts were treated with 2% sodium *N*-lauroylsarcosine (SLS) for 1 h at room temperature, and incubated for 16 h at 4°C thereafter. Extracts corresponding to 1–5 μg of total protein were separated by a 12.5% SDS-PAGE gel and subsequently analyzed by Western blot using anti-HA-antibody (1:300, Roche) and corresponding secondary antibody conjugated with horseradish peroxidase.

### Fast protein liquid chromatography protein purification

Soluble fractions of cell extracts from recombinant strains expressing epitope-tagged proteins were diluted with buffer containing 20 mM NaH_2_PO_4_, 0.5 M NaCl, pH 7.4 to 1% sodium *N*-lauroylsarcosine and loaded on a HisTrap™ HP column (GE Healthcare) previously equilibrated with buffer containing 20 mM NaH_2_PO_4_, 0.5 M NaCl, 0.2% sodium *N*-lauroylsarcosine and 20 mM imidazole, pH 7.4. Proteins were eluted applying an imidazole gradient (0.125-0.5 M). As a further purification step, if necessary, HisTrap™ HP column flow through was dialyzed against buffer containing 20 mM Tris-hydroxymethyl-aminomethane, 0.1 M NaCl, 0.1 mM EDTA, pH 7.5 and loaded onto anti-HA-affinity matrix (Roche). Proteins were eluted with buffer containing 0.1 M glycine, pH 2.0.

### MALDI-TOF/TOF analysis

100–200 pmol of purified lipoprotein were prepared and analyzed according to Ujihara et al. [35]. Briefly, lipoproteins in elution fractions from FPLC or HA chromatography were precipitated and SDS-PAGE gel was performed. Proteins separated by electrophoresis were visualized with copper staining. Protein bands with the apparent molecular weight of apolipoprotein/mature lipoprotein were cut from the stained gel. Lipoproteins were in-gel digested with Trypsin or AspN and extracted peptides were dried and dissolved in 5 μl 0.1% trifluoroacetic acid, 50% acetonitrile. Samples were loaded onto the target and covered with 1 μl matrix solution (5 mg ml^-1^ α-cyano-4-hydroxy-cinnamic acid (Bruker Daltonics) in 0.1% trifluoroacetic acid, 50% acetonitrile). The MALDI-TOF/TOF mass spectra were recorded on an Ultraflex II MALDI-TOF/TOF instrument with smartbeam laser upgrade (Bruker Daltonics). The laser was set to a repetition rate of 100 Hz and the ion acceleration voltage was 29.5 kV. The mass measurements were performed in the positive ion reflector mode.

## Results

### Lipoproteins are expressed in *M. bovis BCG*

As model substrates for lipoprotein modification in slow-growing mycobacteria we chose four different lipoproteins being identical in *M. tuberculosis* and in *M. bovis* BCG Pasteur. The well characterized LppX [12,36] and LprF [13] in addition to LpqH and LpqL. LppX (Rv2945c) has been shown to be involved in translocation of phthiocerol dimycocerosates (DIM) to the outer membrane [36]. LprF (Rv1368) is involved in signaling and has been suggested to interact with the histidine kinase KdpD in response to environmental osmotic stress [37]. LpqH (19 kDa antigen, Rv3763) functions as an adhesin and has been recognized as an immunodominant lipoprotein [38]. LpqL (Rv0418) is predicted to be a lipoprotein aminopeptidase. Hence, our choice of lipoproteins is representing different classes of lipoproteins. The four expression vectors pMV261-Gm for hexa-histidine/hemagglutinine tagged LprF, LpqH, LpqL or LppX were transformed into *M. bovis* BCG. Whole cell extracts from the four strains expressing the recombinant lipoproteins were analyzed by Western blot. The apparent molecular masses of the detected proteins correspond to the predicted mass of the recombinant apolipoproteins/mature lipoproteins (LprF 29.4 kDa, LpqH 17.3 kDa, LpqL 54.2 kDa, LppX 26.3 kDa). Eventually the prepro-/pro-lipoprotein forms whose sizes are increased by 2–3 kDa due to the presence of the signal peptide, are also detected.

### Identification of the lipoprotein lipid anchor in *M. bovis BCG*

To characterize the modifications of lipoproteins at the molecular level, the four recombinant lipoproteins LprF, LpqH, LpqL and LppX were expressed in *M. bovis* BCG parental strain. Proteins were purified by FPLC or HA affinity chromatography. Eluted fractions were analyzed by Western blot (see Additional file 1) and lipoprotein containing fractions were precipitated for SDS-PAGE gel. Bands of purified lipoproteins were visualized with copper staining, cut from the gel and the proteins were in-gel digested with Trypsin or AspN (in case of LprF). Resulting peptides were prepared and analyzed by MALDI-TOF/TOF mass spectrometry [35]. For the identification of the modification we determined the structure and calculated the expected monoisotopic molecular masses of the unmodified N-terminal tryptic or AspN-digested peptides of LprF, LpqH, LpqL and LppX (without signal peptide). Phospholipids found in mycobacteria mainly consist of palmitic (C16:0), palmitoleic (C16:1), oleic (C18:1) and tuberculostearic acid (10-methyloctadecanoic acid) (C19:0) [39]. In *E. coli*, fatty acids of membrane phospholipids, i.e. myristic (C14:0), palmitic, palmitoleic, oleic (C18:1 ω9) or vaccenic (18:1 ω7) acid are used for the modification of lipoproteins [40-44]. Therefore we calculated the theoretical mass of the N-terminal peptides of the four lipoproteins with all possible combinations of the above mentioned fatty acids observed in mycobacterial phospholipids to identify putative modifications. Glycosylations are also commonly found in lipoproteins [45,46]. Some of the analyzed N-terminal peptides carry putative *O*-glycosylation sites, therefore we also calculated the masses with hexose modifications. [M+H]^+^ signals at *m/z* values which we calculated for the unmodified N-terminal peptides were not found. Instead, we found MS signals at *m/z* values which indicate that the N-terminal peptides are modified in a lipoprotein-specific manner with different combinations of saturated and unsaturated C16, C18 and C19 fatty acids. The calculated *m/z* values are summarized and compared with the experimentally determined *m/z* values in Table 1.

**Table 1:** **Comparison of *m/z *values of N-terminal AspN-digested/tryptic peptides of LprF, LpqH, LpqL and LppX found in BCG parental and Δ*lnt *mutant strain**

	**Peptide**	**Calculated *m/z***	**Parental strain *m/z***	***Δlnt m/z***
**LprF**	**CGK…ILQ**	**2496.24**	-	-
	**CGK…ILQ**	3047.11	-	3046.70
	+ Diacylglycerol (C16/C16)	(+550.87)		(+550.46)
	**CGK…ILQ**	3073.15	-	3072.71
	+ Diacylglycerol (C16/C18)	(+576.91)		(+576.47)
	**CGK…ILQ**	3089.20	-	3088.74
	+ Diacylglycerol (C16/C19)	(+592.96)		(+592.50)
	**CGK…ILQ**	3251.44	-	3251.65
	+ Diacylglycerol (C16/C19)	(+755.20)		(+755.41)
	+ Hexose			
	**CGK…ILQ**	3327.60	3326.83	-
	+ Diacylglycerol (C16/C19)	(+831.36)	(+830.59)	
	+ N-acyl (C16)			
	**CGK…ILQ**	3531.93	3530.56	**-**
	+ Diacylglycerol (C16/C19)	(+1035.69)	(+1034.32)	
	+ N-acyl (C19)			
	+ Hexose			
**LpqH**	**CSSNK**	**538.23**	-	-
	**CSSNK**	1089.10	-	1088.60
	+ Diacylglycerol (C16/C16)	(+550.87)		(+550.37)
	**CSSNK**	1115.14	-	1114.68
	+ Diacylglycerol (C16/C18)	(+576.91)		(+576.45)
	**CSSNK**	1131.19	1130.79	1130.71
	+ Diacylglycerol (C16/C19)	(+592.96)	(+592.56)	(+592.48)
	**CSSNK**	1369.59	1369.04	-
	+ Diacylglycerol (C16/C19)	(+831.36)	(+830.81)	
	+ N-acyl (C16)			
**LpqL**	**CIR**	**391.21**	-	-
	**CIR**	984.17	984.50	983.77
	+ Diacylglycerol (C16/C19)	(+592.96)	(+593.29)	(+592.56)
	**CIR**	1222.57	1221.98	-
	+ Diacylglycerol (C16/C19)	(+831.36)	(+830.77)	
	+N-acyl (C16)			
**LppX**	**CSS…EIR**	**2964.46**	-	-
	**CSS…EIR**	3515.33	3514.94	3514.94
	+ Diacylglycerol (C16/C16)	(+550.87)	(+550.48)	(+550.48)
	**CSS…EIR**	3557.42	-	3556.96
	+ Diacylglycerol (C16/C19)	(+592.96)		(+592.50)
	**CSS…EIR**	3719.66	**-**	3719.00
	+ Diacylglycerol (C16/C19)	(+755.20)		(+754.54)
	+Hexose			
	**CSS…EIR**	3795.82	3795.21	-
	+ Diacylglycerol (C16/C19)	(+831.36)	(+830.75)	
	+ N-acyl (C16)			
	**CSS…EIR**	3881.90	-	3881.06
	+ Diacylglycerol (C16/C19)	(+917.44)		(+916.60)
	+ 2 Hexoses			
	**CSS…EIR**	3958.06	3957.28	-
	+ Diacylglycerol (C16/C19)	(+993.60)	(+992.82)	
	+ N-acyl (C16)			
	+ Hexose			
	**CSS…EIR**	4120.30	4119.45	**-**
	+ Diacylglycerol (C16/C19)	(+1155.84)	(+1154.99)	
	+ N-acyl (C16)			
	+ 2 Hexoses			

Peptides correspond to the N-terminal AspN-digested/tryptic peptides of LprF, LpqH, LpqL and LppX upon cleavage of the signal peptide by LspA. Mass differences to the corresponding unmodified peptide (bold number) due to modifications are given in parentheses. Observed modifications are: diacylglycerol with C16 fatty acid and C16 fatty acid (+550.87 Da). Diacylglycerol with C16 fatty acid and tuberculostearic acid (C19:0) (+592.96 Da), plus one hexose (+162.24 Da, Σ = 755.20 Da) or two hexoses (+324.48 Da, Σ = 917.44). Diacylglycerol with C16 fatty acid and C19:0 fatty acid (+592.96 Da) plus *N*-acyl with C16 fatty acid (+238.40 Da, Σ = 831.36), *N*-acyl with C16 fatty acid plus one hexose (+162.24 Da, Σ = 993.6 Da) or two hexoses (+324.48 Da, Σ = 1155.84 Da). Or diacylglycerol with C16 fatty acid and C19:0 fatty acid (+592.96 Da) plus *N*-acyl with C19:0 fatty acid and hexose (+280.49 Da +162.24 Σ = 1035.69).

The modifications we estimated from the [M+H]^+^ signals in the MS spectrum were confirmed by MS/MS fragmentation and thereby information about the linkage of the modification was obtained. The structures of the di- or triacylated N-terminal tryptic or AspN-digested peptides from LprF, LpqH, LpqL and LppX were investigated by MS/MS. All eliminations found in MS/MS of lipoproteins isolated from the parental strain are summarized in Table 2.

**Table 2:** **Comparison of experimentally determined eliminations from N-terminal AspN digested/tryptic peptides of LprF, LpqH, LpqL and LppX in the MALDI-TOF/TOF spectra of BCG parental and Δ*lnt *mutant strain with theoretically calculated eliminations**

**Modification**	**Eliminated fragment**	**Calculated mass of eliminated fragment [ *Da *]**	**Experimentally determined mass of eliminated fragment [ *Da *]**											
			***Parental strain***					**Δ*lnt***						
			**LprF**		**LpqH**	**LpqL**	**LppX**	**LprF**		**LpqH**			**LpqL**	**LppX**
			**C16/C19 C16**	**C16/C19 C19**	**C16/C19 C16**	**C16/C19 C16**		**C16/C16**	**C16/C19**	**C16/C16**	**C16/C18**	**C16/C19**	**C16/C19**	
*O*-linked palmitoyl (C16)	Palmitic acid	**256.24**	256.5	-	256.3	256.3	n.d. *	-	-	256.2	256.1	256.3	256.3	n.d. *
*O*-linked oleyl (C18)	Oleic acid	**282.24**	-	-	-	-	n.d. *	-	-	-	282.4	-	-	n.d. *
*O*-linked tuberculostearyl (C19)	Tuberculostearic acid	**298.29**	-	-	298.3	298.3	n.d. *	-	-	-	-	298.3	298.4	n.d. *
*N-*linked palmitoyl (C16) + Didehydroalanine	Palmitamide + Didehydroalanine	**307.26**	-	306.6	-	-	n.d. *	-	-	-	-	-	-	n.d. *
*N*-linked tuberculostearyl (C19) + Didehydroalanine	Tuberculostearinamide + Didehydroalanine	**349.31**	349.8	-	-	-	n.d. *	-	-	-	-	-	-	n.d. *
Diacylglyceryl (C16/C16)	Diacylhioglyceryl (C16/C16)	**584.44**	-	-	-	-	n.d. *	583.3	-	-	-	-	-	n.d. *
Diacylglyceryl (C16/C18)	Diacylhioglyceryl (C16/C18)	**610.52**	-	-	-	-	n.d. *	-	-	-	-	-	-	n.d. *
Diacylglyceryl (C16/C19)	Diacylhioglyceryl (C16/C19)	**626.53**	625.9	626.7	626.7	626.6	n.d. *	-	626.7	-	-	626.6	626.7	n.d. *
	C16 fatty acid α-thioglyceryl ester	**328.24**	-	-	328.4	328.3	n.d. *	-		-	-	-	-	n.d. *
	C19 fatty acid α-thioglyceryl ester	**370.29**	-	-	370.5	370.3	n.d. *	-	369.8	-	-	-	370.4	n.d. *
Hexose	Hexose	**160.76**	161.62	-	-	-	n.d. *	-	162.9	-	-	-	-	n.d. *

* MALDI-TOF/TOF data for LppX from *M. bovis* BCG were not determined, since MS data of LppX from this study are comparable with data of LppX from *M. smegmatis* (A. Tschumi et al. 2009).

### Lipoproteins in slow-growing Mycobacteria are *N*-acylated with C16 or C19 fatty acids

Since *N*-acylation was shown to be a common motif in lipoproteins of high GC-rich Gram-positive *M. smegmatis*[12,13]*,* we proposed Lnt modification also taking place in slow-growing mycobacteria. This proposal was based on the observation that *M. tuberculosis* apolipoprotein *N*-acyltransferase Ppm1 could complement a *M. smegmatis lnt* mutant [12].

In *M. bovis* BCG, differences in molecular mass of about 831.36 Da for LprF, LpqH, LpqL and LppX, 993.60 Da for LppX, 1035.69 Da for LprF and 1155.84 Da for LppX between the experimentally determined peptide and unmodified N-terminal peptide were found (Table 1). These differences indicated posttranslational modifications of lipoproteins by Lgt, LspA and Lnt. The difference in molecular mass of 831.36 Da points to a modification with diacylglyceryl residue with ester-linked C16 and C19 fatty acid and amide-linked C16 fatty acid. The difference of 993.60 Da indicates a modification with diacylglyceryl residue with ester-linked C16 and C19 fatty acid, amide-linked C16 fatty acid and a glycosylation with one hexose on an *O*-glycosylation site in the N-terminal peptide of LppX. The difference of 1155.84 Da points to a modification with diacylglyceryl residue carrying ester-linked C16 and C19 fatty acid, amide-linked C16 fatty acid and a glycosylation with two hexoses. The difference in molecular mass of 1034.32 Da suggests a modification of LprF with diacylglyceryl residue carrying ester-linked C16 and C19 fatty acid, amide-linked C19 fatty acid and a glycosylation with one hexose (Table 1). Moreover, differences in molecular mass of about 550.87 Da for LppX and 592.96 Da for LpqH, LpqL and LppX were found, both indicating (Lgt and LspA, but not Lnt modified peptides carrying) a diacylglycerol modification with ester-linked C16 and C16 or ester-linked C16 and C19 fatty acid, respectively.

All modifications we estimated from MS data were confirmed by MS/MS (Table 2). Modifications with diacylglyceryl residue were confirmed by eliminations of fragments with the mass of 626.53 Da (C16/C19), corresponding to the elimination of a diacylthioglyceryl carrying C16 and C19 fatty acid. The *O*-linked C16 or C19 fatty acids were confirmed by neutral losses of 256.24 Da and 298.29 Da, corresponding to the elimination of palmitic acid or tuberculostearic acid. Further, neutral losses of 328.24 Da and 370.29 Da correspond to the elimination of C16 or C19 fatty acid α-thioglyceryl ester, respectively. Proposed modification with *N*-linked C16 fatty acid was identified by the neutral loss of 307.26 Da which is consistent with the elimination of palmitamide plus didehydroalanine. Glycosylations in the tryptic or AspN-digested N-terminal peptides at other amino acids than the conserved cysteine were confirmed by the eliminations of fragments of 162.24 Da for each hexose. (Note, since MS data of LppX from this study are comparable with data from our recent study in *M. smegmatis*[12], MS/MS data for LppX were not further determined).

**Figure 1:**
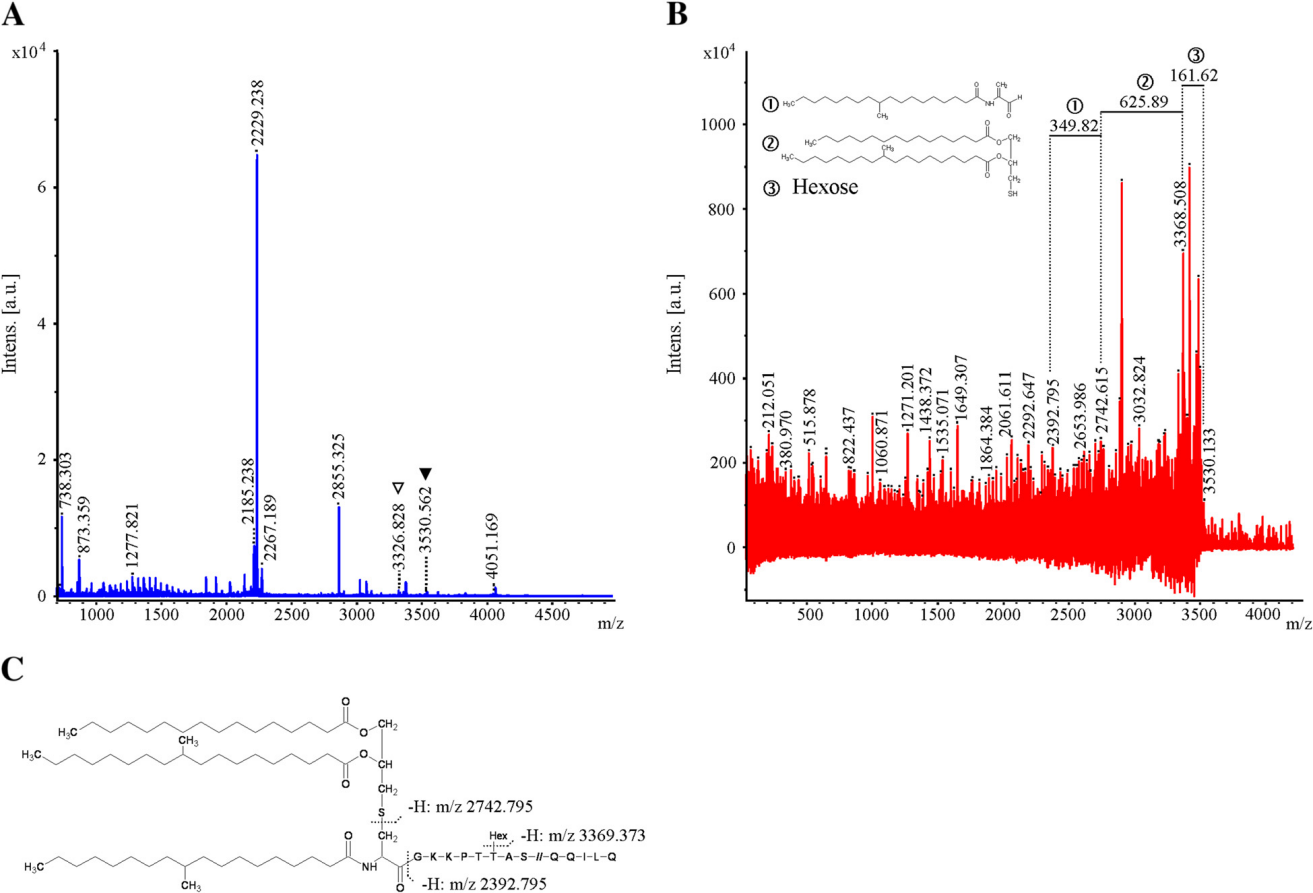
**MALDI-TOF and MALDI-TOF/TOF analysis of the N-terminal peptides of LprF. A**. MS analysis of AspN-digested peptides of LprF purified from *M. bovis* BCG parental strain. *Filled triangle*, diacylglycerol (C16/C19) + *N*-acyl (C16) modified and glycosylated N-terminal peptide, *open triangle,* diacylglycerol (C16/C19) + *N*-acyl (C19) modified and glycosylated N-terminal peptide **B**. MS/MS analysis of the N-terminal peptide of LprF from *M. bovis* BCG parental strain. Eliminated fragments of LprF modifications are shown in the upper part of the spectrum. ➀ Tuberculostearinamide + Didehydroalanine, ➁ Diacylthioglyceryl (C16/C19), ➂ Hexose. **C**. Schematic drawing of the modified +1 cysteine with the cleavage sites of each identified *m/z* signal.

Previous structure analyses of lipoprotein modifications in *M. smegmatis* recovered C16 and C19 moieties as ester-linked acyl residues of the diacylglycerol and C16 fatty acid exclusively as substrate for *N*-acylation [12,13]. However, beside the signal at m/z = 3326.828, an additional signal at m/z = 3530.562 was found in the MS of LprF (Figure 1A). The signal at m/z = 3326.828 corresponds to LprF modified with a diacylglyceryl residue carrying ester-linked C16 and C19 fatty acid and *N*-linked C16 fatty acid. Eliminated fragments in MS/MS analysis of the signal m/z = 3530.562 (Figure 1B) confirmed a modification with diacylglyceryl residue carrying ester-linked C16 and C19 fatty acid, *N*-linked C19 fatty acid and a hexose. The neutral loss of 625.89 Da from the ion at m/z = 3368.508 corresponds to the elimination of diacylthioglyceryl carrying both *O*-linked C16 and C19 fatty acids. In addition, the neutral loss of 349.82 Da from m/z = 2742.615 corresponds to the elimination of tuberculostearinamide plus didehydroalanine. This fragmentation pattern shows that the +1 cysteine is modified at the sulfhydryl group by a diacylglyceryl residue carrying ester-bound C16 fatty acid and C19 fatty acid and an amide-bound C19 fatty acid at the cysteine (Figure 1C).

### Generation of an *lnt* deletion mutant in *M. bovis BCG*

**Figure 2:**
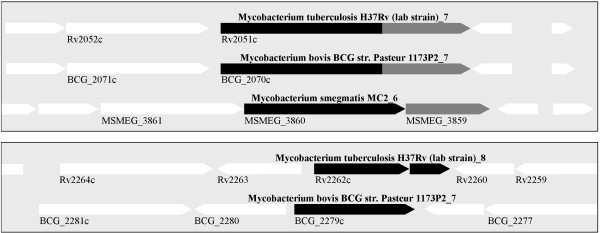
**A comparison of the genomic region of Lnt homologues in mycobacteria.** Black bars/arrows indicate Lnt homologues. A second domain is fused to the *lnt* domain in *M. tuberculosis* Rv2051c, and *M. bovis* BCG BCG_2070c (grey arrows) and is homologous to *M. smegmatis* MSMEG_3859 (grey arrow). White arrows indicate orientation of surrounding genes.

Using *E. coli* Lnt as a query in a BLASTp search on a subset of mycobacteria, we identified three open reading frames annotated as polyprenol-monophosphomannose synthase Ppm1, i.e. Rv2051c in *M. tuberculosis*, BCG_2070c in *M. bovis* BCG Pasteur and MSMEG_3860 in *M. smegmatis*, respectively. In *M. tuberculosis* two additional putative homologous open reading frames, Rv2262c and Rv2261c annotated as hypothetical proteins were found (Figure 2). Both, MSMEG_3860 as well as the N-terminal part of the two-domain protein encoded by Rv2051c are already identified as functional *N*-acyltransferases in mycobacteria [12]. A further search with *M. tuberculosis* Rv2262c/2261c as a query in a BLASTp search identified BCG_2279c as homologue in *M. bovis* BCG Pasteur, whereas no homologue was found in *M. smegmatis*. We used sequence alignment with the Needleman-Wunsch algorithm (http://www.ebi.ac.uk/Tools/psa/emboss_needle) with default settings to compare both *M. bovis* ORFs to *E. coli lnt*, *M. tuberculosis lnt* Rv2051c, as well as *M. tuberculosis* Rv2262c/2261c sequences. Pairwise sequence alignment revealed the highest sequence identity (100%) between BCG_2070c and Rv2051c from *M. tuberculosis*. Interestingly, pairwise sequence alignment of BCG_2279c and Rv2262c/2261c reveals that both sequences differ by a 2 bp insertion in Rv2262c (see Additional file 2). This leads to a stop codon and initiation of Rv2261c with codon ttg. BCG_2279c does not have this insertion and therefore encodes only one protein. We confirmed this polymorphism by sequencing corresponding regions of *M. tuberculosis* and *M. bovis* BCG genomes. We also used protein sequence alignment with the Needleman-Wunsch algorithm (http://www.ebi.ac.uk/Tools/psa/emboss_needle)  and ClustalW2 (http://www.ebi.ac.uk/Tools/msa/clustalw2/) with default settings to analyze the conservation of essential residues (see Additional file 3). BCG_2070c and Rv2051c showed conservation of 14 among 23 residues required for optimal activity of *E. coli* Lnt and conservation of the three essential residues of the catalytic triad of *E. coli* Lnt i.e. E267, K335, C387 (see Additional file 4) [11]. For comparison, the alignment of BCG_2279c and Rv2262c/2261c with *E. coli* Lnt also showed conservation of 13 or 12 (in Rv2262c/2261c *E. coli* P346 is altered from proline to leucine) among the 23 residues of *E. coli* Lnt. However, different residues among the 23 were conserved (see Additional file 4). In BCG_2279c and Rv2262c/2261c it revealed that essential residue C387 of the catalytic triad is altered from cysteine to serine. C387 is essential for Lnt-activity and transfer of the acyl residue to the apo-lipoprotein in *E. coli.* However, it has been shown that a Lnt (C387S) mutant also formed an acyl-enzyme intermediate as the wildtype Lnt C387, but did not have any detectable Lnt activity [11,47]. Moreover, it is noteworthy that the residues of the catalytic triad are separated on two different ORFs encoded by Rv2262c/2261c in *M. tuberculosis*. Beside the three essential residues of the catalytic triad, four other essential residues W237, E343, Y388 and E389 are absolutely required for Lnt function. Among these seven essential residues, five residues are conserved in *M. tuberculosis* Rv2051c, Rv2262c/2261c and *M. bovis* BCG BCG_2070c, BCG_2279c Lnt homologues.

In summary, homology searches and comparison of essential residues in the putative Lnts revealed only small differences and it may be hypothesized that both BCG_2070c and BCG_2279c are functional *N*-acyltransferases. BCG_2070c is identical to an ORF with proven *N*-acyltransferase activity since *M. tuberculosis* Lnt complemented the *M. smegmatis lnt* deletion mutant and all three residues of the catalytic triad essential for Lnt function in *E. coli* are conserved. Lnt activity of BCG_2279c may be buried by the Lnt activity of BCG_2070c. Therefore we generated a BCG_2070c *lnt* deletion mutant and characterized lipoprotein modifications in the mutant. The *lnt* deletion mutant was constructed by transformation of *M. bovis* BCG with the suicide plasmid pMCS5-*rpsL-hyg-*Δ*lnt*BCG applying *rpsL* counter-selection strategy, a powerful tool to generate deletion mutants in mycobacteria [31,32]. The mutant strain resulting from allelic exchange is referred to as *M. bovis* BCG Δ*lnt*. Deletion of *lnt* was verified by Southern blot analysis using a 5’*lnt* DNA probe (see Additional file 5). The probe hybridized to an 8.1-kbp fragment of the parental strain and to a 3.1-kbp fragment of the Δ*lnt* mutant. Moreover, a complemented mutant strain was constructed by transformation of *M. bovis* BCG Δ*lnt* mutant with complementation vector pMV361-hyg-*lnt*BCG_2070c expressing *M. bovis* BCG BCG_2070c. The complemented strain is referred to as *M. bovis* BCG Δ*lnt*-*lnt*BCG_2070c.

### BCG_2070c is a functional *N*-acyltransferase in *M. bovis BCG*

The four expression vectors pMV261-Gm for hexa-histidine/hemagglutinine tagged LprF, LpqH, LpqL or LppX were transformed into *M. bovis* BCG Δ*lnt* mutant. Recombinant lipoproteins expressed in the four strains were analyzed by Western blot. The apparent molecular masses of the detected proteins correspond to the predicted mass of the recombinant apolipoproteins/mature lipoproteins. Eventually the prepro-/pro-lipoprotein forms, whose sizes are increased by 2–3 kDa due to the presence of the signal peptide, are also detected.

In order to characterize BCG_2070c and eventually residual *N*-aclytransferase activity in *M. bovis BCG*, lipoprotein modifications of LprF, LpqH, LpqL and LppX from Δ*lnt* mutant were analyzed at the molecular level. In Δ*lnt*, signals with molecular masses indicating Lgt- and LspA- modified and glycosylated peptides were found. The differences in molecular mass of 550.87 Da for LprF, LpqH and LppX and 576.91 Da for LprF and LpqH between the experimentally found peptide and the unmodified N-terminal peptide (Table 1) indicate (Lgt and LspA, but not Lnt modified peptides carrying) a diacylglycerol modification carrying ester-linked C16 and C16 or ester-linked C16 and C18 fatty acid, respectively. The differences in molecular mass of 592.96 Da for LprF, LpqH, LpqL and LppX refer to a diacylglycerol modification with ester-linked C16 and C19 fatty acid. The differences in molecular mass of 755.20 Da for LprF and LppX refer to a diacylglycerol modification with ester-linked C16 and C19 fatty acid plus glycosylation with one hexose (592.96 Da + 162.24 Da). The difference in molecular mass of 917.90 Da for LppX refers to a diacylglycerol modification with ester-linked C16 and C19 fatty acid plus modification with two hexoses (592.96 Da + 162.24 Da + 162.24 Da).

In contrast to the MS from parental strain, no molecular masses which we calculated for modifications with three fatty acids were found in the Δ*lnt* mutant strain. In particular, the differences in molecular mass of 238.4 Da (831.36 Da - 592.96 Da) or 280.49 Da (1035.69 Da - 162.24 Da - 592.96 Da) between the C16/C19/C16 or C16/C19/C19 triacylated modification found in the parental strain and the corresponding estimated C16/C19 modification in the Δ*lnt* mutant indicate a lack of *N*-acylation with a C16 or C19 fatty acid in the Δ*lnt* mutant. In MS/MS analysis, this indication of missing *N*-acylation in the mutant was confirmed by identification of the estimated modifications and information about its linkage (Table 2). Modifications with C16/C19 diacylglyceryl residue were confirmed by eliminations of fragments with the molecular mass of 626.53 Da, corresponding to the elimination of a diacylthioglyceryl carrying C16 and C19 fatty acid. The *O*-linked C16 or C19 fatty acids were confirmed by neutral losses of 256.24 Da or 298.29 Da, corresponding to the elimination of palmitic acid or tuberculostearic acid, respectively. Further, the neutral loss of 370.29 Da corresponds to the elimination of C19 fatty acid α-thioglyceryl ester. A glycosylation at other amino acids than the conserved cysteine was confirmed by the release of a fragment of 162.24 Da for a hexose. These findings indicate that *N*-acylation is not a prerequisite for glycosylation. As mentioned before, only diacylglyceryl residues composed of a C16 and a C19 fatty acid were identified in mycobacterial lipid anchors so far [12,13]. However, the eliminations of fragments with the molecular mass of 584.44 Da or 256.24 Da, corresponding to the elimination of diacylthioglyceryl and palmitic acid, give evidence for modifications with diacylglyceryl residue carrying C16 and C16 fatty acids. Moreover, estimated diacylglycerol modifications carrying C16 and C18 fatty acids were confirmed by neutral losses of fragments with the molecular mass of 256.24 Da and 282.44 Da, corresponding to the elimination of palmitic and oleic acid. In complemented mutant Δ*lnt*-*lnt*BCG_2070c, lipoproteins LprF and LppX were triacylated and glycosylated (see Additional files 6 and 7). This confirmed that BCG_2070c restored the BCG_2070c mutant.

The absence of *N*-acylation of the four analyzed lipoproteins in the Δ*lnt* mutant and the complementation of the mutant provide strong evidence that BCG_2070c is the only functional apolipoprotein *N*-acyltransferase that modifies these lipoproteins with an amide-linked fatty acid in *M. bovis* BCG. In addition, it demonstrates that BCG_2279c is not able to adopt or substitute *N*-acylation of the four lipoproteins in the Δ*lnt* mutant.

## Discussion

Lipoproteins are present in all bacterial species, but their biogenesis and lipid moieties differ, especially between Gram-negative and Gram-positive bacteria. The three enzymes involved in lipoprotein biosynthesis, namely Lgt, LspA and Lnt first were identified in *E. coli*. Therefore, the lipoprotein biosynthesis pathway in *E. coli* is intensively studied and well described [6]. Mycobacteria are classified as Gram-positive bacteria, but their lipoprotein biosynthesis pathway resembles that of Gram-negative bacteria. The discovery of Lnt in mycobacteria and the identification of lipoprotein *N*-acylation in *M. smegmatis* renewed interest within the field of mycobacterial lipoprotein research. The evidence of triacylated lipoproteins in mycobacteria refuted the long held assumption, that *N*-acylation is restricted to Gram-negative bacteria. Thus, the acylation with three fatty acids is a common feature of mycobacterial and *E. coli* lipoproteins. But, mycobacterial lipoproteins differ from *E. coli* lipoproteins with respect to the fatty acids used for the triacylation. Mycobacteria-specific fatty acid 10-methyl octadecanoic acid (tuberculostearic acid) is uniquely found in lipoproteins of *M. smegmatis*[12,13].

All three enzymes of the lipoprotein biosynthesis pathway, Lgt, LspA and Lnt are essential in Gram-negative, but not in Gram-positive bacteria. However, in *M. tuberculosis, lgt*, the first enzyme of the lipoprotein biosynthesis pathway is essential. A targeted deletion of *lgt* was not possible [48]. In contrast, an *lspA* deletion mutant was viable, but the mutant strain showed a reduced number of CFU in an animal model and induced hardly any lung pathology. This confirmed a role of the lipoprotein biosynthesis pathway in pathogenesis of *M. tuberculosis*[23,24].

Lipoproteins itself are well known virulence factors in pathogenic bacteria. *M. tuberculosis* lipoproteins in particular have been shown to suppress innate immune responses by TLR2 agonist activity [26]. Newest data also show that lipoproteins constitute the main proteinaceous content of membrane vesicles released by pathogenic mycobacteria and that they are highly immunogenic [49]. Several *M. tuberculosis* mutants deficient in individual lipoproteins are attenuated in virulence as shown for LppX [50], LprG [51] and LpqH [52]. Recently, a *M. tuberculosis* deletion mutant, defective in lipoprotein LpqS showed attenuation in macrophages [53]. Despite the important role of *M. tuberculosis* lipoproteins in immunogenicity and pathogenicity and all the achievements in knowledge about the lipoprotein modification in apathogenic *M. smegmatis*, still little is known about the molecular structure of lipoproteins in pathogenic mycobacteria. The elucidation of lipoprotein structure can build the fundamental knowledge for future development of lipoprotein based subunit vaccines and antitubercular drugs targeting enzymes of the lipoprotein synthesis pathway [54]. Therefore we extended our research in lipoprotein modifications to slow-growing mycobacteria. Most of the pathogenic mycobacteria and the tuberculosis vaccine strain *M. bovis* BCG belong to this sub-group.

In the present study, we investigated the lipid moieties of four mycobacterial lipoproteins representing lipoproteins with different functions. By MALDI-TOF/TOF analyses of a Trypsin digest of purified LpqH, LpqL and LppX and an AspN digest of purified LprF, we unambiguously identified modifications at the universally conserved cysteine in the parental strain. All four proteins were found to be triacylated carrying a thioether-linked diacylglyceryl residue with C16 and C19 fatty acid (C16/C19) to the sulfhydryl group of the lipobox cysteine and an amide-linked C16 fatty acid. Whether the fatty acids of the diacylglyceryl residue are in the *S*_n_1 or *S*_n_2 position could not be determined by mass spectrometry and therefore currently remains elusive. In LprF, a novel triacylation with C16/C19 diacylglycerol and C19 *N*-acyl was identified. This differs from previous lipoprotein analyses in *M. smegmatis*, where C16 fatty acid was the single substrate for Lnt [12,13]. Likewise, it shows that mycobacteria not only use mycobacteria-specific fatty acids for diacylglycerol modification, but also for *N*-acylation. Lipoprotein modifications with acyl residues of different length, stiffness and bulkiness may influence membrane fluidity and localization of lipoproteins. In *Francisella novicida*, an environmentally regulated membrane remodelling directed by multiple alleles of the lipid A-modifying *N*-acyltransferase enzyme is reported. By incorporation of shorter or longer *N*-acyl fatty acid chains to the outer membrane lipid A, the bacterium regulates the maintenance of membrane fluidity and integrity [55]. Therefore, it is obvious to speculate a similar important role of the C19 *N*-acyl lipoprotein modification for mycobacteria in terms of adaptations to environmental alterations or specific bacterial conditions. Adaptation of lipoprotein modification to environmental conditions has been shown for *S. aureus*. A combination of conditions including acidic pH and post-logarithmic growth phase induced the accumulation of diacylated lipoproteins [56].

By the usage of C19 fatty acid, mycobacterial Lnt strongly differs in substrate specificity from *E. coli* Lnt. *E. coli* Lnt utilizes all three major phospholipids of *E. coli* phosphatidylethanolamine, phosphatidylglycerol and cardiolipin as its fatty acid source in vivo [40]. Subsequent analysis revealed that both the phospholipid head group and its acyl chain composition affect *N*-acyltransferase activity in vitro [41]. *E. coli* Lnt incorporates palmitic (C16) fatty acids from the *S*_*n*_1 position of phospholipids to diacylated lipoproteins [42]. In mycobacterial phospholipids the *S*_*n*_1 position is esterified principally with octadecanoic or tuberculostearic acid (C18 related fatty acids), whereas palmitic acid (C16) is mainly located at the *S*_*n*_2 position [57]. Based on this and the fact, that palmitic acids were used for *N*-acylation of lipoproteins in *M. smegmatis*[12,13], Nakayama et al. proposed that *M. smegmatis* Lnt uses fatty acids from the *S*_*n*_2 position as substrates and therefore has a different specificity than *E. coli* Lnt [20]. This specificity obviously is different in *M. bovis* BCG. Our results provide strong evidence, that not only palmitic acid from the *S*_*n*_2 position, but also tuberculostearic acid (C19), a fatty acid from the *S*_*n*_1 position of phospholipids is transferred by Lnt [57].

Lipoproteins are recognized by TLR2 in association with TLR1 or TLR6. While diacylated lipoproteins carrying the *S*-diacylglyceryl residue are recognized by TLR2/6 heterodimers, triacylated lipoproteins carrying the additional *N*-acyl are recognized by TLR1/2 heterodimers. The two ester-bound fatty acids are inserted into a pocket in TLR2 while the amide-bound fatty acid is inserted into a hydrophobic channel in TLR1. Therefore the *N*-acyl of the lipoprotein is indispensable for the heterodimerization of TLR2 and TLR1 and thus the initiation of TLR2/1 signaling [58,59]. Recent investigations indicate that TLR1 polymorphisms are associated with resistance towards bacterial pathogens, including *M. tuberculosis*[60,61]. It may be hypothesized that the modification of lipoproteins with particular fatty acids plays a crucial role for lipoprotein function, its retention in a membrane, and interaction with TLRs. However, whether the *N*-acylation with C19 fatty acid is only characteristic for LprF or also for other lipoproteins and whether it is a feature of *M. bovis* BCG Lnt remains to be investigated.

Beside the triacylated forms, also diacylated forms of the N-terminal peptide were found in proteins from the parental BCG strain. A modification with C16/C19 diacylglycerol was found in LpqL and a C16/C16 diacylglycerol was found in LppX. These molecules probably indicate N-terminal peptides from unmature proteins which have not been converted to mature lipoproteins by Lnt yet.

Lipoproteins from *M. bovis* BCG were identified to be triacylated at their N-terminus which suggests that *N*-acylation by an Lnt enzyme also exists in slow-growing mycobacteria. We identified the open reading frame, encoding the Lnt enzyme responsible for the *N*-acylation. *M. bovis* BCG Pasteur genome analysis revealed two open reading frames BCG_2070c and BCG_2279c homologous to *E. coli* Lnt. Our biochemical analyses of four lipoproteins expressed in a BCG_2070c Δ*lnt* mutant demonstrated that BCG_2070c is the major if not the only functional mycobacterial Lnt in *M. bovis* BCG. When we subjected lipoproteins LprF, LpqH, LpqL and LppX expressed in the Δ*lnt* mutant to MALDI-TOF/TOF analyses, none of the proteins was found to be *N*-acylated. All four proteins were found to be only diacylated in contrast to the triacylated proteins in the parental strain. Diacylglyceryl residues composed of C16/C19 fatty acid, C16/C16 fatty acid or C16/C18 were found. Hereby the usage of oleic acid as a substrate for lipoprotein modification in mycobacteria, to our knowledge is shown for the first time.

We showed that the lack of BCG_2070c results in a failure of lipoprotein *N*-acylation and that BCG_2279c is not able to compensate Lnt function. BCG_2279c has a C to S amino acid substitution in C387, a residue essential for Lnt function in *E. coli*. In *E. coli*, a C387 alteration absolutely abolishes Lnt function, because this residue is part of the catalytic triad of Lnt [11]. Alterations in BCG_2279c therefore could account for its inactivity as Lnt. But we cannot exclude that BCG_2279c is a second Lnt particularly active under specific growth conditions. Alternatively, BCG_2279c may act only on a small subset of dozens of putative mycobacterial lipoproteins not yet characterized by MALDI-TOF/TOF.

*Streptomyces spp.*, bacteria closely related to mycobacteria, also encode two Lnt homologues. Deleting *Streptomyces scabies lnt1* and *lnt2* genes individually or in combination revealed that Lnt1 is a functional Lnt sufficient and required for *N*-acylation. Lnt2 could not compensate for the Lnt1 deletion. However, both Lnts seem to be required for efficient lipoprotein *N*-acylation as the lack of Lnt2 alone resulted in a marginal *N*-acylation activity. This implies a subsidiary but inessential role for Lnt2, not directly involved in *N*-acylation of lipoproteins [15]. Likewise, an interplay can count for the two Lnt homologues in *M. bovis* BCG. But, in contrast to the Lnts in *S. scabies*, BCG_2279c is missing one of the three essential residues required for Lnt activity in *E. coli.* This, in our opinion diminishes the possibility for BCG_2279c to be an Lnt with *N*-acylation activity and favours a contributive role for it. In vitro biochemical assays [41] with purified BCG_2279c or analyses of a BCG_2279c mutant alone or in combination with BCG_2070c would be required to elucidate this.

Beside the fatty acid modifications, we also identified hexose glycosylations in LprF and LppX. Modifications with one or more glycosyl residues have been shown for several mycobacterial lipoproteins [13,45,62]. *O*-glycosylation occurs at Ser and Thr residues respectively. Although glycosylations of the tryptic or AspN-digested N-terminal peptides of LprF and LppX were identified, the exact glycosylation site within the peptide could not be determined. No glycosylations were found for N-terminal fragments of LpqH and LpqL. This possibly is due to the use of proteases which have cleavage sites close to the N-terminus and therefore the peptide fragment may be too short to include *O*-glycosylation sites. The information about the exact molecular nature and function of the glycosylation is scarce, but its influence on subcellular lipoprotein localization and its protection from proteolytic degradation are proposed [45,62]. In *B. subtilis* lipoprotein glycosylation is discussed to control a lipoprotein “shaving” mechanism and thus their release into the culture medium [63]. In our study, glycosylations were found also in lipoproteins from the Δ*lnt* mutant, demonstrating that *N*-acylation is not a prerequisite for glycosylation. Lnt independent glycosylation was also demonstrated in *C. glutamicum*[16]. In *C. glutamicum* Cg-Ppm1 is responsible for glycosylation. Cg-ppm1 (Ppm synthase) and Cg-ppm2 (Lnt) are similar organized as MSMEG_3859 (Ppm synthase) and MSMEG_3860 (Lnt) in *M. smegmatis* (Figure 2). Deletion of the Lnt domain of BCG_2070c obviously did not abolish Ppm activity encoded in the same ORF. Of note, Lnt is dispensable while Ppm is essential in *M. tuberculosis*[64].

In Gram-negative bacteria, the efficient lipoprotein transport to the outer membrane depends on the localization of lipoproteins (Lol) transport system and there is good evidence that *N*-acylation by Lnt facilitates lipoprotein translocation in *E. coli*[6,65]. Lnt is essential in *E. coli*, however deletion of *lnt* was possible upon overexpression of proteins from the Lol system, indicating an important role of *N*-acylation in targeting lipoproteins to the outer membrane [9]. Mycobacteria have an outer membrane mycolic acid bilayer [66-68] and are known to localize lipoproteins to the cell surface [66]. Nevertheless, no mechanisms for translocation or transport systems are identified and whether *N*-acylation and glycosylation, alone or in combination are involved in the translocation of specific lipoproteins to the mycolate layer is not known so far.

In the present study we show that lipoproteins from *M. bovis* BCG, the live vaccine for tuberculosis are triacylated and we identified the lipid modifications at the molecular level. BCG_2070c is a functional homologue of *E. coli* Lnt, but differs in substrate specificity. The identification of *N*-linked tuberculostearic acid shows for the first time, to our knowledge, that mycobacteria-specific fatty acids are used by mycobacterial Lnts.

The antituberculosis drug pipeline is not sufficiently filled and the vaccines used at present do not provide effective protection against tuberculosis in adults. For lipoproteins and their biosynthesis pathway potential implications in *M. tuberculosis* pathogenesis and immunogenicity have been shown. Our results about lipoprotein structure therefore may contribute to provide the knowledge which is required to develop novel vaccines and antituberculosis drugs to eliminate this worldwide epidemic.

## Conclusions

Lipoproteins are triacylated in slow-growing mycobacteria. By MALDI-TOF/TOF analyses lipoprotein modifications in *M. bovis* BCG wildtype and BCG_2070c *lnt* deletion mutant were analyzed at the molecular level. *N*-acylation of lipoproteins was only found in the wildtype strain, but not in the mutant strain, which confirmed BCG_2070c as functional *lnt* in *M. bovis* BCG. Moreover, we identified mycobacteria-specific tuberculostearic acid as further substrate for *N*-acylation in slow-growing mycobacteria.

## Competing interests

The authors declare that they have no competing interests.

## Authors’ contributions

JKB designed the study, performed experimental work and drafted the manuscript. AT carried out the genetic engineering of *M. bovis* BCG mutant strain and participated in the MS/MS data analyses. PS conceived of the study, participated in its coordination and helped to draft the manuscript. All authors read and approved the final manuscript.

## Supplementary Material

Additional file 1: Figure S1 Western blot analysis of purified lipoproteins of *M. bovis* BCG wildtype and Δ*lnt* mutant strain. Click here for file

Additional file 2: Figure S2 Sequence alignment of *M. tuberculosis* Rv2262c/Rv2261c and *M. bovis* BCG_2070c using EMBOSS Needle. Click here for file

Additional file 3: Figure S3 Multiple sequence alignment of Lnt homologues using Clustal W2. Click here for file

Additional file 4: Table S1 Conservation of essential residues in Lnt homologues. Click here for file

Additional file 5: Figure S4 Disruption of *Mycobacterium bovis* BCG *lnt* (BCG_2070c). Click here for file

Additional file 6: Figure S5 MALDI-TOF analysis of the N-terminal peptides of LprF. Click here for file

Additional file 7: Figure S6 MALDI-TOF analysis of the N-terminal peptides of LppX. Click here for file
