# Supplementary material for: Lipoproteins of slow-growing Mycobacteria carry three fatty acids and are N-acylated by Apolipoprotein N-Acyltransferase BCG_2070c
Source: BMC Microbiol. 2013 Oct 5;13:223. doi: 10.1186/1471-2180-13-223 (PMC3850990; doi:10.1186/1471-2180-13-223)
Supplement: Additional file 1: Figure S1 — Western blot analysis of purified lipoproteins of M. bovis BCG wildtype and Δlnt mutant strain. [file 1471-2180-13-223-S1.doc]

**Supplemental Figure S1**


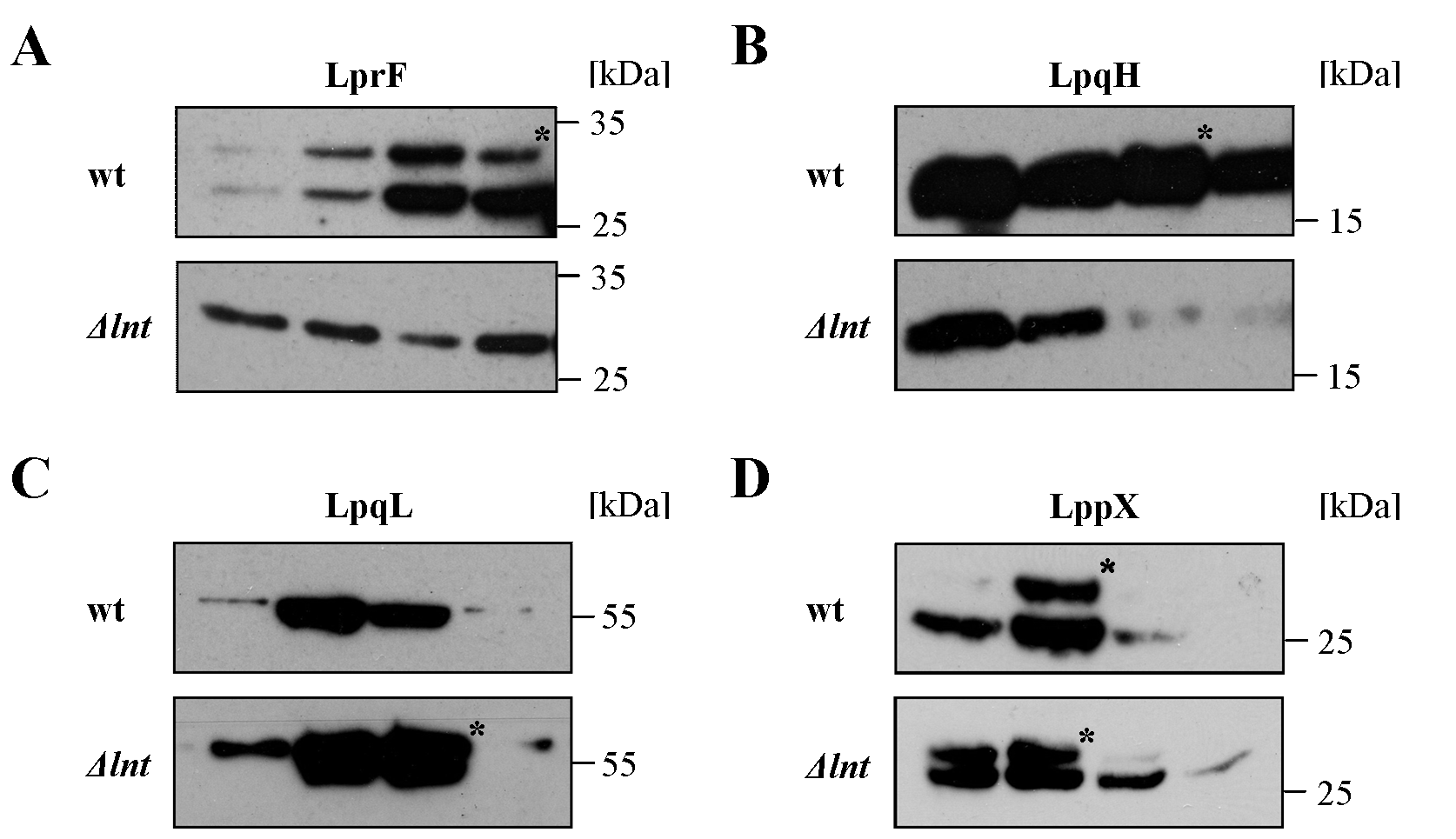


**Fig. S1. Western blot analysis of purified lipoproteins of *M. bovis* BCG wildtype and Δ*lnt* mutant strain.** Elution fractions from FPLC or HA affinity chromatography were analyzed by Western blot using anti-HA antibody and corresponding secondary antibody conjugated with horseradish peroxidase. A. LprF B. LpqH C. LpqL D. LppX. Bands with a higher molecular weight (labeled with an asterisk) correspond to pre-pro-lipoprotein or pro-lipoprotein, lower bands correspond to apolipoprotein and/or mature lipoprotein, respectively.
