# Supplementary material for: Lipoproteins of slow-growing Mycobacteria carry three fatty acids and are N-acylated by Apolipoprotein N-Acyltransferase BCG_2070c
Source: BMC Microbiol. 2013 Oct 5;13:223. doi: 10.1186/1471-2180-13-223 (PMC3850990; doi:10.1186/1471-2180-13-223)
Supplement: Additional file 2: Figure S2 — Sequence alignment of M. tuberculosis Rv2262c/Rv2261c and M. bovis BCG_2070c using EMBOSS Needle. [file 1471-2180-13-223-S2.doc]

**Supplemental Figure S2**


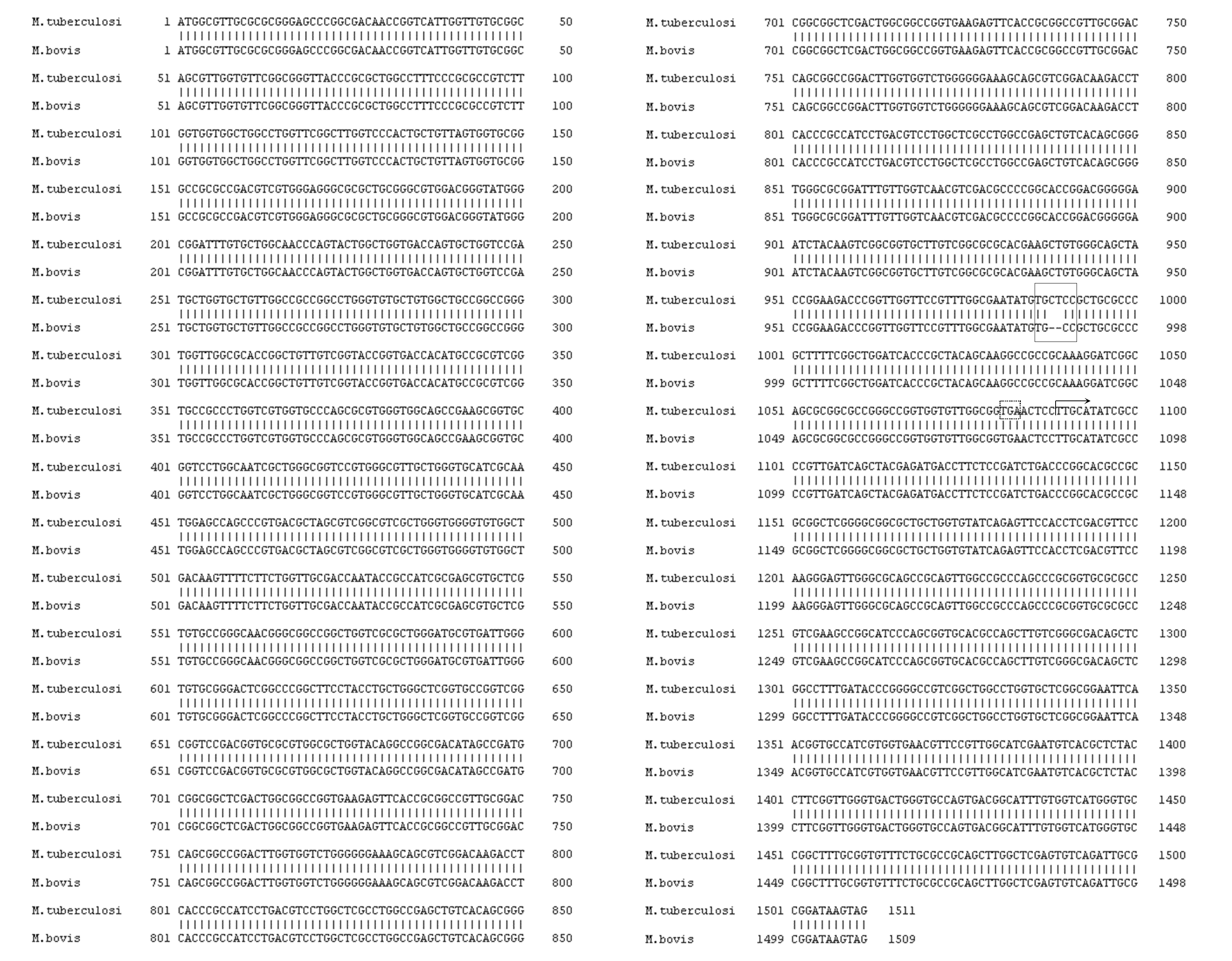


**Fig. S2. Sequence alignment of *M. tuberculosis* Rv2262c/Rv2261c and *M. bovis* BCG_2070c using EMBOSS Needle.** 2bp difference in sequence is marked with a box. Resulting stop codon TAG of Rv2262c is marked with a box (dotted line) and start of Rv2261c is indicated with an arrow.
