# Supplementary material for: Lipoproteins of slow-growing Mycobacteria carry three fatty acids and are N-acylated by Apolipoprotein N-Acyltransferase BCG_2070c
Source: BMC Microbiol. 2013 Oct 5;13:223. doi: 10.1186/1471-2180-13-223 (PMC3850990; doi:10.1186/1471-2180-13-223)
Supplement: Additional file 3: Figure S3 — Multiple sequence alignment of Lnt homologues using Clustal W2. [file 1471-2180-13-223-S3.doc]

**Supplemental Figure S3**


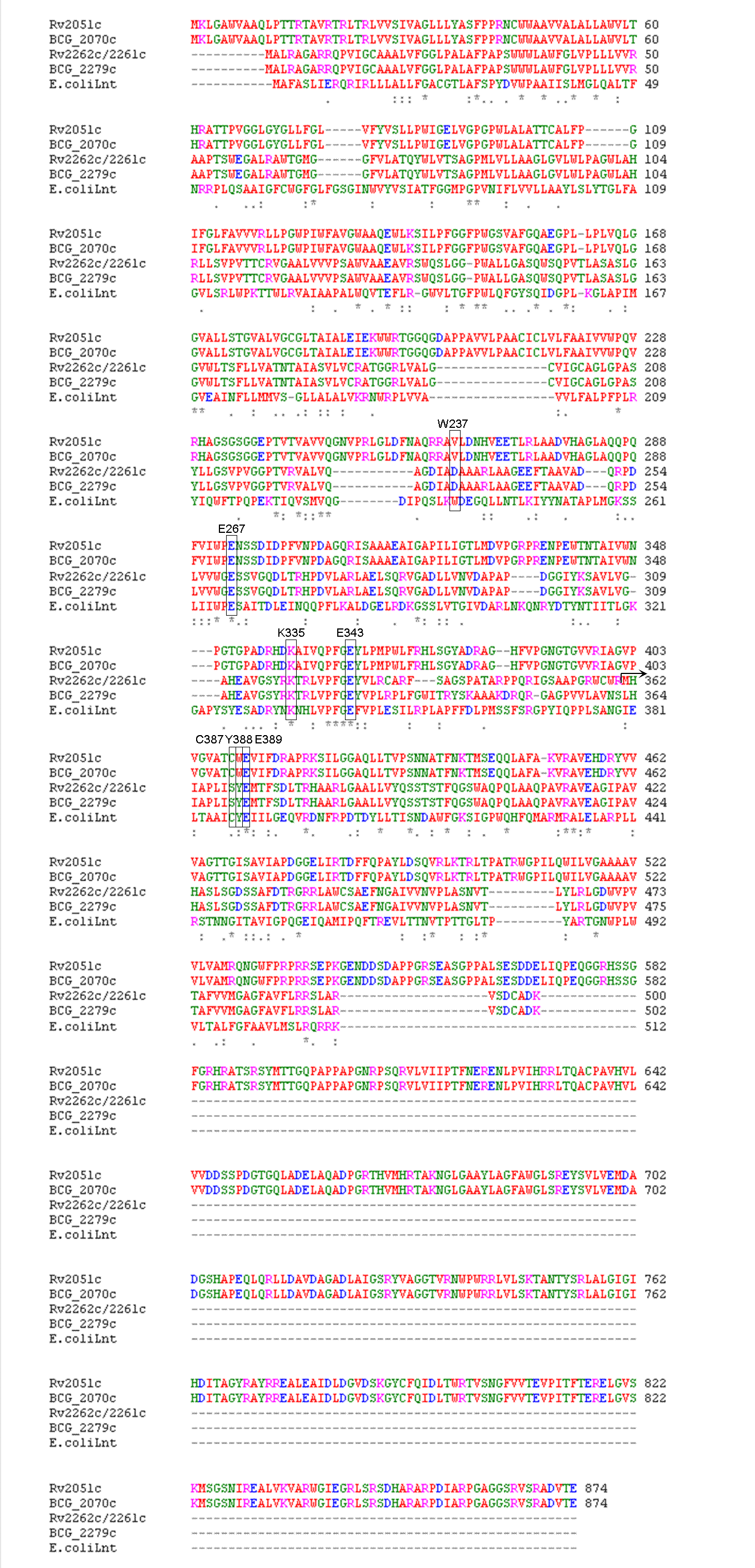


**Fig. S3. Multiple sequence alignment of Lnt homologues using Clustal W2.** Essential residues required for *E. coli* Lnt function and corresponding residues in the Lnt homologues are marked with a box. Start of Rv2261c sequence is indicated with an arrow.
