# Supplementary material for: Lipoproteins of slow-growing Mycobacteria carry three fatty acids and are N-acylated by Apolipoprotein N-Acyltransferase BCG_2070c
Source: BMC Microbiol. 2013 Oct 5;13:223. doi: 10.1186/1471-2180-13-223 (PMC3850990; doi:10.1186/1471-2180-13-223)
Supplement: Additional file 4: Table S1 — Conservation of essential residues in Lnt homologues. [file 1471-2180-13-223-S4.doc]

**TABLE S1. Conservation of essential residues in Lnt homologues.**

| ***Escherichia coli***  **Lnt**  **essential residue*** | ***Mycobacterium tuberculosis***  **Rv2051c** | ***Mycobacterium bovis* BCG**  **BCG_2070c** | ***Mycobacterium tuberculosis***  **Rv2262c/2261c**** | | | ***Mycobacterium bovis* BCG**  **BCG_2279c** |
| --- | --- | --- | --- | --- | --- | --- |
| W74 | F | F | F | | | F |
| F146 | G | G | G | | | G |
| W148 | + | + | + | | | + |
| Q228 | + | + | + | | | + |
| Q233 | + | + | G | | | G |
| *W237* | V | V | D | | | D |
| Y249 | A | A | T | | | T |
| P266 | + | + | G | | | G |
| **E267** | **+** | **+** | **+** | | | **+** |
| N314 | T | T | K | | | K |
| Y333 | H | H | + | | | + |
| **K335** | **+** | **+** | **+** | | | **+** |
| *E343* | + | + | + | | | + |
| P346 | + | + | L | | | **+** |
| Q372 | T | T |  | A |  | P |
| **C387** | **+** | **+** | **S** | | | **S** |
| *Y388* | W | W | + | | | + |
| *E389* | + | + | + | | | + |
| Q424 | + | + | + | | | + |
| R432 | + | + | + | | | + |
| A433 | + | + | + | | | + |
| E435 | + | + | + | | | + |
| L436 | H | H | A | | | A |

Essential residues of the catalytic triad are written in bold letters. Other essential residues absolutely required for function are written in italic letters.

*Vidal-Ingigliardi *et al.* 2007

**Residues above the bar are encoded by Rv2262c, residues below the bar are encoded by Rv2261c.
