# Supplementary material for: Lipoproteins of slow-growing Mycobacteria carry three fatty acids and are N-acylated by Apolipoprotein N-Acyltransferase BCG_2070c
Source: BMC Microbiol. 2013 Oct 5;13:223. doi: 10.1186/1471-2180-13-223 (PMC3850990; doi:10.1186/1471-2180-13-223)
Supplement: Additional file 5: Figure S4 — Disruption of Mycobacterium bovis BCG lnt (BCG_2070c). [file 1471-2180-13-223-S5.doc]

**Supplemental Figure S4**

**
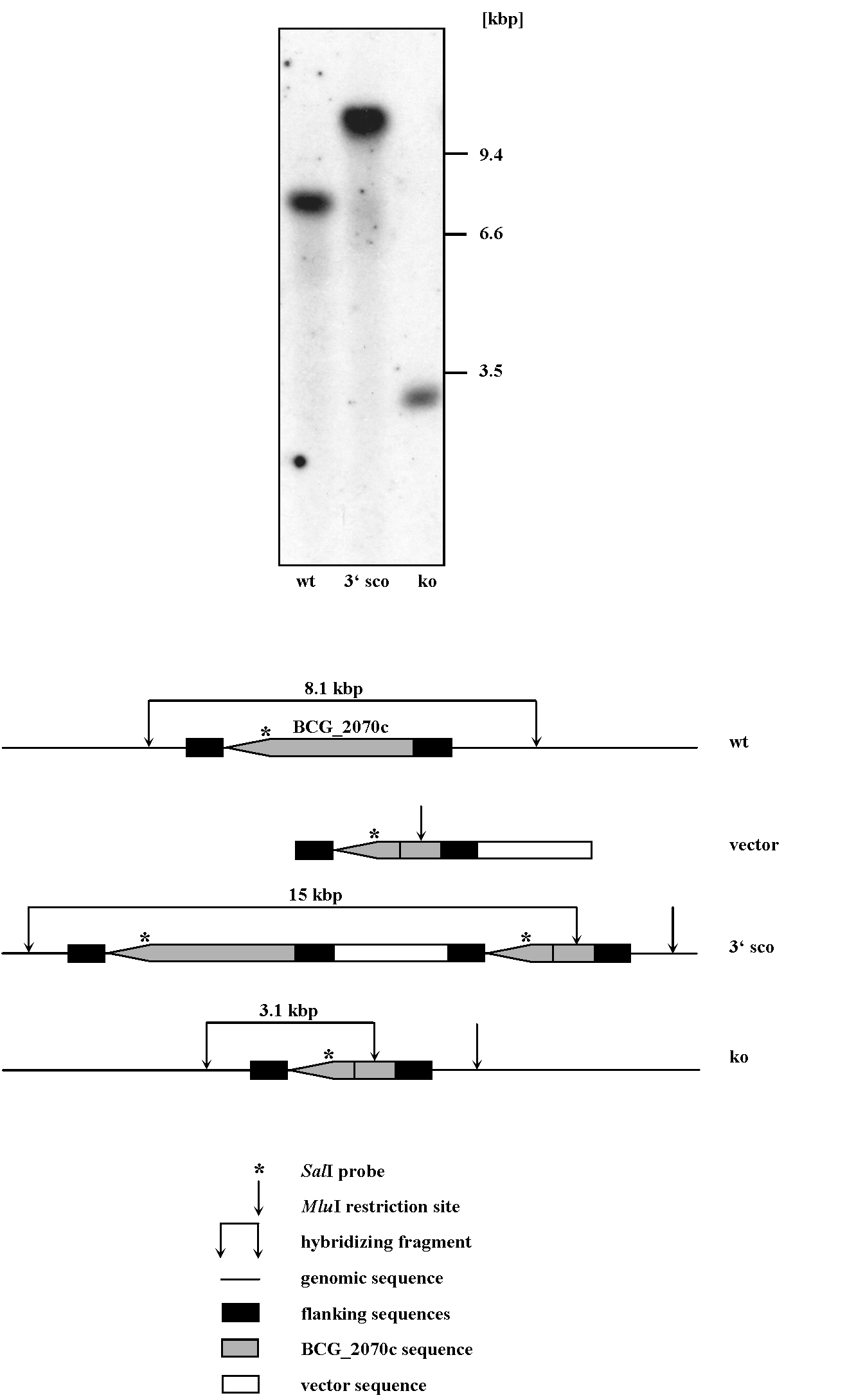
**

**Fig. S4. Disruption of *Mycobacterium bovis* BCG *lnt* (BCG_2070c).** Genomic DNAs from *M. bovis* BCG (lane 1), *lnt* single-crossover (3‘ sco) mutant (lane 2) and Δ*lnt* mutant (lane 3) were digested with *Mlu*I and probed with a 222 bps *Sal*I *lnt* gene fragment. The presence of the 3.1 kbp fragment in the Δ*lnt* knockout compared to the 8.1 kbp fragment in the parental strain demonstrates inactivation of *lnt*.
