# Supplementary material for: Lipoproteins of slow-growing Mycobacteria carry three fatty acids and are N-acylated by Apolipoprotein N-Acyltransferase BCG_2070c
Source: BMC Microbiol. 2013 Oct 5;13:223. doi: 10.1186/1471-2180-13-223 (PMC3850990; doi:10.1186/1471-2180-13-223)
Supplement: Additional file 7: Figure S6 — MALDI-TOF analysis of the N-terminal peptides of LppX. [file 1471-2180-13-223-S7.doc]

**Supplemental Figure S6**


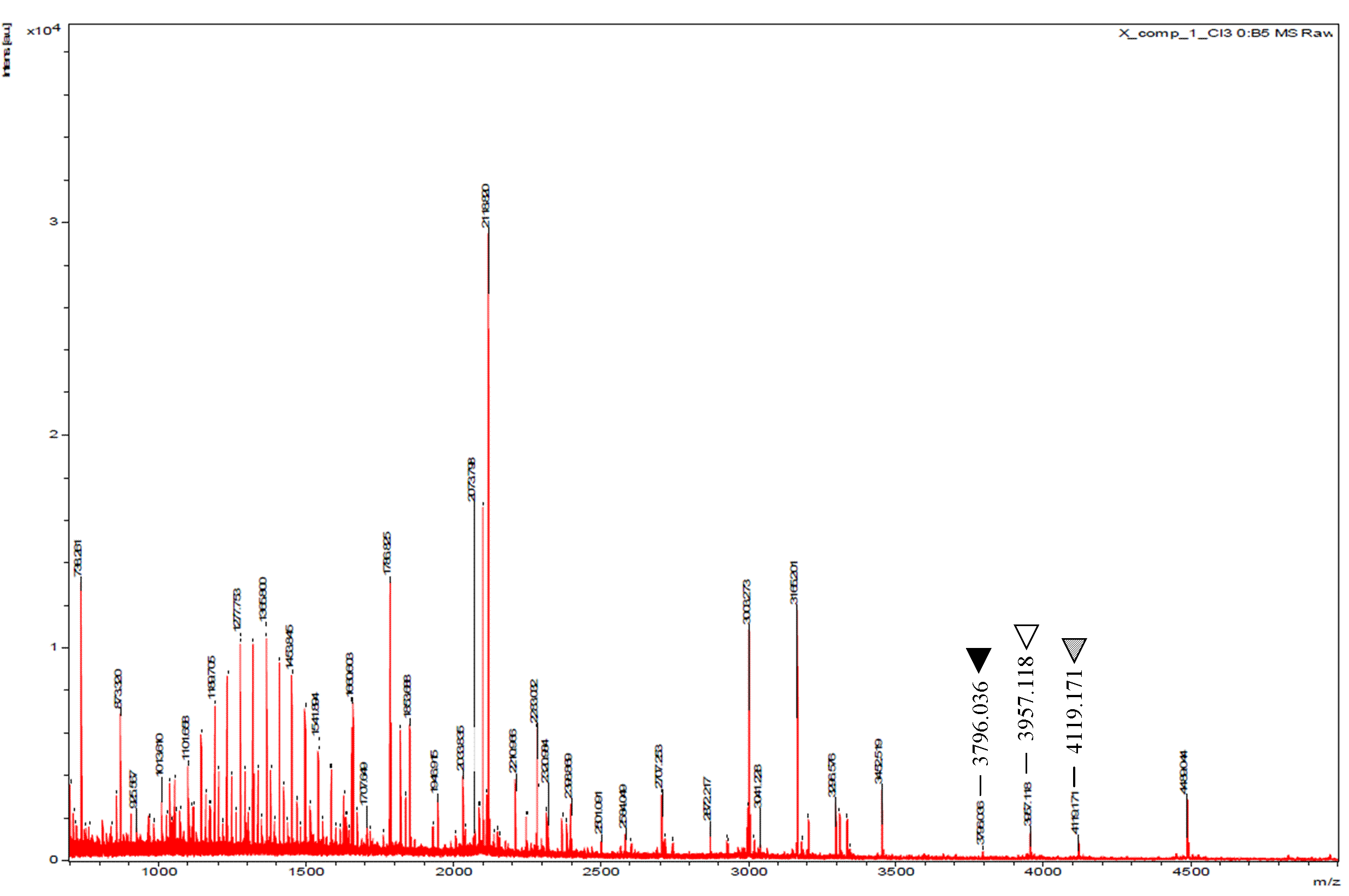


**Fig. S6. MALDI-TOF analysis of the N-terminal peptides of LppX.** MS analysis of Trypsin-digested peptides of LppX purified from complemented mutant *Δlnt-lnt*BCG_2070c. *Filled triangle*, diacylglycerol (C16/C19) + *N*-acyl (C16) modified N-terminal peptide, *open triangle*, diacylglycerol (C16/C19) + *N*-acyl (C16) modified and glycosylated N-terminal peptide, *shaded triangle*, diacylglycerol (C16/C19) + *N*-acyl (C16) modified and with two hexoses glycosylated N-terminal peptide.
